# Metabolic Profiles in Ovine Carotid Arteries with Developmental Maturation and Long-Term Hypoxia

**DOI:** 10.1371/journal.pone.0130739

**Published:** 2015-06-25

**Authors:** Ravi Goyal, Lawrence D. Longo

**Affiliations:** Center for Perinatal Biology, Department of Basic Sciences, School of Medicine, Loma Linda University, Loma Linda, California, United States of America; University of Nebraska Medical Center, UNITED STATES

## Abstract

**Background:**

Long-term hypoxia (LTH) is an important stressor related to health and disease during development. At different time points from fetus to adult, we are exposed to hypoxic stress because of placental insufficiency, high-altitude residence, smoking, chronic anemia, pulmonary, and heart disorders, as well as cancers. Intrauterine hypoxia can lead to fetal growth restriction and long-term sequelae such as cognitive impairments, hypertension, cardiovascular disorders, diabetes, and schizophrenia. Similarly, prolonged hypoxic exposure during adult life can lead to acute mountain sickness, chronic fatigue, chronic headache, cognitive impairment, acute cerebral and/or pulmonary edema, and death.

**Aim:**

LTH also can lead to alteration in metabolites such as fumarate, 2-oxoglutarate, malate, and lactate, which are linked to epigenetic regulation of gene expression. Importantly, during the intrauterine life, a fetus is under a relative hypoxic environment, as compared to newborn or adult. Thus, the changes in gene expression with development from fetus to newborn to adult may be as a consequence of underlying changes in the metabolic profile because of the hypoxic environment along with developmental maturation. To examine this possibility, we examined the metabolic profile in carotid arteries from near-term fetus, newborn, and adult sheep in both normoxic and long-term hypoxic acclimatized groups.

**Results:**

Our results demonstrate that LTH differentially regulated glucose metabolism, mitochondrial metabolism, nicotinamide cofactor metabolism, oxidative stress and antioxidants, membrane lipid hydrolysis, and free fatty acid metabolism, each of which may play a role in genetic-epigenetic regulation.

## Introduction

Metabolomics is the study of biochemical processes that involve cellular metabolites, with an emphasis to identify the unique chemical fingerprint of a given cell under specific, well-defined conditions. Analysis of a cellular metabolic profile is being used in many aspects of biology to help define phenotype, to understand pathways of signal transduction, and to characterize specific cellular aspects of development and disease. The goal of such analysis is to allow more precise definition of the cellular state that will enhance diagnosis and/or the choice of therapy. Of importance, metabolomics is being used to map metabolic pathways under a wide variety of circumstances [1,2]; including studies of biomarkers of normal and complicated pregnancies [3,4], and preterm labor and delivery [5,6]

Long-term hypoxia (LTH) is an important factor in a number of disorders during different phases of organismal life [7–11]. For the developing fetus, it is already in a relatively severe hypoxic environment i.e. “Mount Everest in utero” [12,13]. A further increase in hypoxic stress may lead to growth restriction [14,15] and other life long sequelae [9,16–19]. Similarly in the adult, LTH as a result of high altitude residence can lead to acute/chronic mountain sickness, acute cerebral edema, and/or other conditions [20]. LTH also is an important factor in the pathogenesis of debilitating fatigue and morbidity associated with chronic lung and heart disorders such as chronic obstructive pulmonary disease, pulmonary dysplasia, congestive cardiac failure, and chronic anemia. Thus, it is important to elucidate the cellular and molecular mechanisms regulated by LTH and their relation to developmental age.

In previous studies, we have demonstrated that with LTH, during both fetal and adult life, the carotid arteries play an important role in maintaining cerebral blood flow (CBF) and oxygenation [21–23]. To maintain adequate brain oxygenation under hypoxic stress the carotid arteries in both fetus and adult undergo significant changes in gene expression that affects both their phenotype as well as function [24]. Moreover, carotid arteries have been shown to play a crucial role in the regulation and maintenance of CBF [25]. During increased flow demand, there is a significant pressure gradient from CA to cerebral arteries [26]. Importantly, other studies suggest that much of the change in systemic pressure results in dilation/contraction of the large arteries that supply the brain [27]. These studies underscore the importance of carotid arteries in the regulation of CBF, and suggest that failure of carotid arteries to effectively regulate the pressure of the blood reaching delicate cerebral arteries may result in their rupture with hemorrhage. Moreover, evidence suggests that large arteries of premature as well as intrauterine growth restricted infants may be unable to regulate effectively their CBF, as opposed to the near-term newborn [28, 29]. Others and we have demonstrated that LTH also leads to significant alteration in transcriptome during fetal and adult life [24, 30–32]. In our genomic studies, we have observed that hypoxia inducible factor 1α (HIF1α), the major molecule implicated in acute (short-term) hypoxia, was no longer upregulated in association with LTH [24], which is in agreement with findings reported by others [33]. Nonetheless, following decades of research the molecular pathways involved in LTH-induced changes in gene regulation remain poorly described.

Furthermore, several metabolites are altered with hypoxic stress such as phospo-enol-pyruvate, lactate, fumarate, 2-oxoglutarate, and succinate, each being an important player in epigenetic programming of the genome [34, 35]. Once regarded as a metabolic waste product, lactate also has emerged as an important regulator of number of pathological and physiological processes [36,37]. Evidence is accumulating that LTH-mediated changes in metabolism may be a major factor in the pathogenesis of many disorders including cancer, pulmonary, and heart disorders. Therefore, it is of vital importance to conduct a comprehensive study on the effects of LTH on metabolic processes during advancing periods of life such as the fetus, newborn infant, and adult. Because of the importance of cerebral oxygenation, we thus tested the hypothesis that LTH differentially regulates metabolic pathways in the cranial arteries at these several developmental ages. In addition, these pathways may play an important role in the acclimatization responses to LTH.

## Methods

### Experimental animals and tissues

All experimental procedures were conducted within the regulations of the Animal Welfare Act, the National Institutes of Health Guide for the Care and Use of Laboratory Animals, the Guidelines of the American Physiological Society, and were approved by the Animal Care and Use Committee of Loma Linda University. Sheep were obtained from Nebeker Ranch Inc. (Lancaster, CA), as previously described [24,38]. For these studies, we used carotid arteries from near-term fetuses (~146 gestation day), newborn lambs (1 day to 5 days old), and adult (~ 2 years old ewes), that either had been maintained near sea level (300 m) or those acclimatized to high altitude (3,801 m, 12,470 ft; Barcroft Laboratory, White Mountain Research Station [WMRS], Bishop, CA) for ~ 110 days immediately before the studies.

For each experiment of the study, eight sheep were used from each experimental group. For the control (normoxic) group, sheep were maintained at the suppliers ranch (Nebeker Ranch Inc.) on alfalfa pellets ad libitum. For the LTH group, at 30 days gestation ewes were transported to the Barcroft Laboratory, WMRS; barometric pressure -480 Torr), where they were kept until 135 days gestation (near term) in an outdoor sheltered pen and were fed with alfalfa pellets ad libitum. Sheep from both groups were kept in natural day-night conditions. In our previous studies we have obtained mean maternal arterial blood gas values from 12 adult sheep (same breed and age as used in the present study) while at WMRS, which were PO2 = 60 ± 5 Torr, PCO2 = 30.0 ± 2.5 Torr, and pH = 7.36 ± 0.06. In contrast normoxic control sheep had PO2 of 100 ± 5 Torr, PCO2 35.2 ± 0.9, pH = 7.44 ± 0.1. With LTH exposure, fetal arterial PO_2_ fell from 29.7 ± 2.1 to 19.1 ± 2.1 Torr. At 135 days gestation, ewes from both groups were transported (6 to 7 h trip) to our laboratory at Loma Linda University. Soon after the arrival to the laboratory, in LTH group a tracheal catheter was placed in the ewe, through which N_2_ flowed at a rate adjusted to maintain its PO_2_ at ~60 Torr, i.e., that at high altitude, until surgeries were conducted for tissue isolation. In both groups, the ewes were anesthetized with thiopental sodium (10 mg.kg^-1^, i.v.), and anesthesia was maintained with inhalation of 1% isoflurane in oxygen throughout surgery. From pregnant ewes, the fetuses were delivered by hysterotomy (Cesarean Section) and carotid arteries were isolated. In a similar manner, carotid arteries also were obtained from the non-pregnant ewes. Following surgeries, the fetuses and ewes were euthanized with an overdose of the proprietary euthanasia solution, Euthasol (pentobarbital sodium 100 mg.Kg^-1^ and phenytoin sodium 10 mg.Kg^-1^; Virbac, Ft. Worth, TX). Studies were conducted on isolated mid-carotid artery segments cleaned of blood, adipose, and loose connective tissue. The arterial segments were snap frozen in liquid nitrogen until further analysis.

### Metabolic Profile

Metabolic profiling was conducted utilizing the commercial services of Metabolon Inc. (Durham, NC). Carotid arteries were homogenized and processed by the Intact Sample Extraction method where the volume of methanol used for extraction was proportionately adjusted to the weight of the sample. Samples were prepared using the automated MicroLab STAR system (Hamilton Robotics Inc. Reno, NV). A recovery standard was added prior to the first step in the extraction process for quality control (QC) purposes. Small molecules bound to protein were recovered by precipitating the proteins with methanol under vigorous shaking for 2 min (Glen Mills GenoGrinder 2000, OPS Diagnostics LLC, Lebanon, NJ) followed by centrifugation. The resulting extract was divided into four fractions: one for analysis by Ultra Performance Liquid Chromatography—Tandem Mass Spectrometer (UPLC-MS/MS) with positive ion mode electrospray ionization, one for analysis by UPLC-MS/MS with negative ion mode electrospray ionization, one for analysis by Gas Chromatography—Mass Spectrometry (GC-MS), and one sample was reserved for backup. To remove the organic solvents, samples were placed briefly on a TurboVap (Biotage LLC, Charlotte, NC). Each sample was frozen and dried under vacuum, then prepared for the applicable instrumental analysis.

### Quality Analysis and Quality Control

Several types of controls were analyzed in concert with the experimental samples (A and B Tables in S1 File): a pooled matrix sample was generated by taking a small volume of each experimental sample (or alternatively, use of a pool of well-characterized human plasma) to serve as a technical replicate throughout the data set; extracted water samples served as process blanks; and a cocktail of QC standards (that were carefully chosen not to interfere with the measurement of endogenous compounds) were added into each sample. These controls allowed instrument performance monitoring and aided chromatographic alignment. Instrument variability was determined by calculating the median relative standard deviation (RSD) for the standard solutions injected into the mass spectrometers. Overall process variability was determined by calculating the median RSD for all endogenous metabolites (i.e., non-instrument standards) present in 100% of the pooled matrix samples. Experimental samples were randomized across the platform run with QC samples spaced evenly among the injections. This technique has been used in several reported studies [39,40].

### Liquid Chromatography-Tandem Mass Spectrometry (LC-MS/MS)

The LC-MS portion of the platform was based on a Waters ACQUITY (Milford, MA) ultra-performance liquid chromatography (UPLC) and a Thermo-Finnigan (Pittsburgh, PA) LTQ mass spectrometer operated at nominal mass resolution, which consisted of an electrospray ionization (ESI) source and linear ion-trap (LIT) mass analyzer. The sample extract was dried then reconstituted in acidic or basic LC-compatible solvents, each of which contained 12 or more injection standards at fixed concentrations. One aliquot of sample was analyzed using acidic positive ion-optimized conditions, and the other sample aliquot was analyzed using basic negative ion-optimized conditions. Both aliquots were analyzed using two independent injections with separate dedicated columns (Waters UPLC BEH C18-2.1×100 mm, 1.7 μm). Extracts reconstituted in acidic conditions were gradient-eluted using water and methanol with 0.1% formic acid, while the basic extracts, used water/methanol with 6.5 mM ammonium bicarbonate. The MS analysis alternated between MS and data-dependent MS/MS scans using dynamic exclusion with the scan range of 80–1000 m/z. Raw data files were archived and extracted, as described below.

### Gas Chromatography-Mass Spectroscopy (GC-MS)

The samples destined for analysis by GC-MS were dried under vacuum for a minimum of 18 h prior to being derivatized under dried nitrogen using bistrimethyl-silyltrifluoroacetamide. Derivatized samples were separated on a 5% diphenyl / 95% dimethyl polysiloxane fused silica column (20 m x 0.18 mm ID; 0.18 um film thickness) with helium as carrier gas and a temperature ramp from 64° to 340°C in a 17.5 min period. Samples were analyzed on a Thermo-Finnigan Trace DSQ fast-scanning single-quadrupole mass spectrometer using electron-impact ionization (EI) and operated at unit mass resolving power. The scan range was from 50 to 750 mass to charge ratio (m/z). Raw data files were archived and extracted as described below.

### Data Extraction and Compound Identification

Raw data was extracted, and the compound peaks were identified by comparison to library entries of purified standards or recurrent unknown entities in the Metabolon Inc Library. This library is based on authenticated standards that contain the retention time/index (RI), m/z, and chromatographic data (including MS/MS spectral data) of major metabolites. Furthermore, biochemical identifications were based on three criteria: 1. RI within a narrow window of the proposed identification, 2. accurate mass match to the library +/- 0.005 atomic mass units, and 3. the MS/MS forward and reverse scores between the experimental data and authentic standards. The MS/MS scores were based on a comparison of the ions present in the experimental spectrum to the ions present in the library spectrum. While there may be similarities between these molecules based on one of these factors, the use of all three data points can be utilized to distinguish and differentiate biochemicals. More than 3300 commercially available purified standard compounds were registered into the laboratory information management system for distribution to both the LC-MS and GC-MS platforms for determination of their analytical characteristics. Additional mass spectral entries were created for structurally unnamed biochemicals, which have been identified by virtue of their recurrent nature (both chromatographic and mass spectral). These compounds have the potential to be identified by future acquisition of a matching purified standard or by classical structural analysis.

### Curation

A variety of curation procedures were carried out to ensure that a high quality data set was made available for statistical analysis and data interpretation. The QC and curation processes were designed to ensure accurate and consistent identification of true chemical entities, and to remove those representing system artifacts, mis-assignments, and background noise. Proprietary visualization and interpretation software (Metabolon Inc.) were used to confirm the consistency of peak identification among the various samples. Library matches for each compound were checked for each sample and corrected if necessary.

### Metabolite Quantification and Data Normalization

Peaks were quantified using area-under-the-curve. As the studies spanned multiple days, a data normalization step was performed to correct variation resulting from the instrument inter-day tuning differences. Essentially, each compound was corrected in run-day blocks by registering the medians to equal one (1.00) and normalizing each datum proportionately (termed the “block correction”). Furthermore, data was normalized to total protein, as determined by Bradford assay, to account for differences in metabolite levels due to differences in the amount of material present in each sample.

### Statistical Methods

Standard statistical analyses were performed in ArrayStudio (OmicSoft Corporation, Cary, NC) on log transformed data. For those analyses not standard in ArrayStudio, the program R (http://cran.r-project.org/) was used. Random Forest analysis was used to classify the metabolic profiles of carotid artery samples from the six experimental groups: 1) to assess the capacity to distinguish between different time points based on global metabolic profiles and 2) to identify biochemicals important to the classification. An accuracy of 50% was expected by random chance when comparing two groups. Two-way ANOVA with contrasts was used to identify biochemicals that differed significantly between experimental groups following log transformation and imputation of missing values, if any, with the minimum observed value for each compound. Analysis by two-way ANOVA identified biochemicals exhibiting significant interaction and main effects for experimental parameters of developmental stage and hypoxia. An estimate of the false discovery rate (*q*-value) was calculated to consider the multiple comparisons that normally occur in metabolomic-based studies. For each group n = 8 and P value <0.05 was considered as a cut-off for significance.

## Results

**Fig 1:**
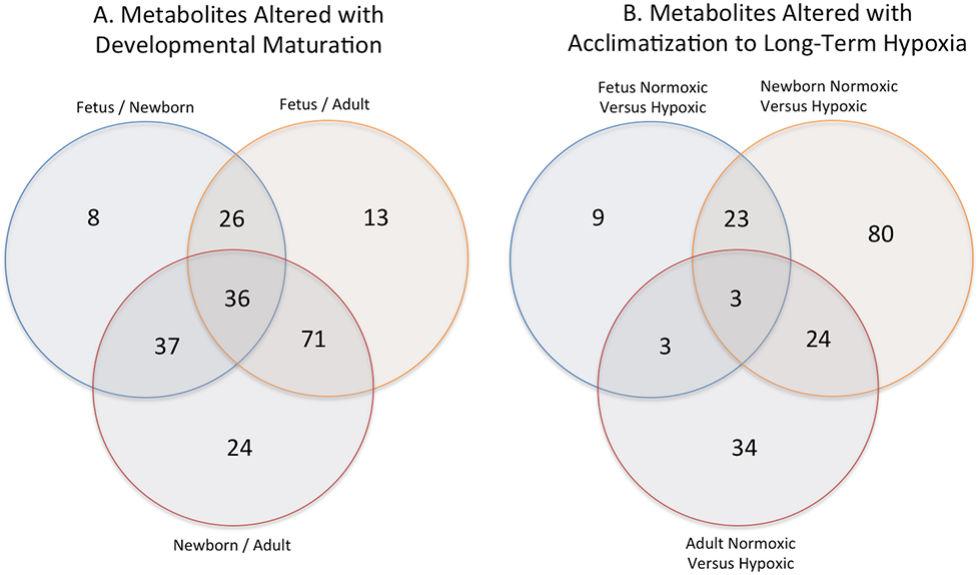
A. Venn diagram of carotid artery metabolites altered with developmental maturation in comparing normoxic fetus and newborn, fetus and adult, and newborn and adult. B. Venn diagram of metabolites altered with long-term hypoxia exposure on comparing normoxic versus hypoxic fetus, newborn, and adult carotid arteries.

The metabolic profile analysis identified a total of 256 compounds (biochemicals) (Table C in S1 File). A summary of the numbers of biochemicals that achieved statistical significance (p≤0.05) is shown in Table 1. Analysis by two-way ANOVA identified biochemicals exhibiting significant interaction and main effects for experimental parameters of developmental stage and hypoxia. Fig 1A and 1B present Venn diagrams that demonstrate the number of metabolites altered with development and hypoxia, respectively.

**Table 1:** Statistical Comparison.

Statistical Comparisons									
ANOVA Contrasts	FH/FN	NBH/NBN	AH/AN	NBN/FN	AN/FN	AN/NBN	NBH/FH	AH/FH	AH/NBH
Total Biochemicals P < 0.05	23	107	39	86	135	150	80	140	87
Biochemicals (upregulated/downregulated)	12/11	62/45	29/10	74/12	108/27	89/61	68/12	117/23	59/28
Total Biochemicals 0.05<p<0.10	15	23	25	20	17	19	20	17	20
Biochemicals (upregulated/downregulated)	12/3	20/3	22/3	14/6	8/9	14/5	17/3	15/2	14/6
Two-Way ANOVA Main Effects	Developmental Stage Main Effect			Hypoxia Main Effect			Developmental Stage: Hypoxia Interaction		
Total Biochemicals P < 0.05	189			115			60		
Total Biochemicals 0.05<p<0.10	12			27			20		

### Overall Changes in metabolic profile with development

Table 2 lists the major metabolites altered with the three developmental maturation stages examined. The major metabolic pathways affected by developmental maturation were those involved in amino acids, peptides, mitochondrial metabolism, carbohydrates, lipids, nucleotide, cofactors and vitamins (Tables 3–7).

**Table 2:** Metabolites altered in all three developmental groups.

Biochemical Name	NBN/FN	AN/FN	AN/NBN
linoleate (18:2n6)	21.83	5.58	0.26
dihomo-linolenate (20:3n3 or n6)	11.35	2.98	0.26
eicosapentaenoate (EPA; 20:5n3)	8.14	3.02	0.37
oleate (18:1n9)	5.96	0.47	0.08
arachidonate (20:4n6)	5.02	2.02	0.4
2-linoleoylglycerol (2-monolinolein)	4.84	9.24	1.91
15-methylpalmitate (isobar with 2-methylpalmitate)	4.45	1.76	0.4
myristoleate (14:1n5)	3.36	0.56	0.17
carnitine	3.14	11.87	3.78
cis-vaccenate (18:1n7)	3.08	0.3	0.1
dihomo-linoleate (20:2n6)	3.07	0.52	0.17
tyrosylglycine	2.97	1.97	0.66
nonadecanoate (19:0)	2.93	1.68	0.57
10-heptadecenoate (17:1n7)	2.81	0.46	0.17
adenosine 3'-monophosphate (3'-AMP)	2.76	0.21	0.08
1-arachidonoylglycerophosphocholine (20:4n6)*	2.64	5.09	1.93
eicosenoate (20:1n9 or 11)	2.52	0.46	0.18
mead acid (20:3n9)	2.46	0.13	0.05
guanosine 3'-monophosphate (3'-GMP)	2.45	0.26	0.11
docosahexaenoate (DHA; 22:6n3)	2.39	0.53	0.22
S-adenosylhomocysteine (SAH)	2.32	4.79	2.07
palmitoleate (16:1n7)	2.3	0.24	0.11
myristate (14:0)	1.91	0.56	0.29
docosatrienoate (22:3n3)	1.9	0.1	0.05
isoleucine	1.85	2.72	1.47
erucate (22:1n9)	1.82	0.29	0.16
1-oleoylplasmenylethanolamine*	1.71	0.47	0.27
methionine	1.61	3.26	2.03
acetylcarnitine	1.6	8.66	5.4
phenylalanine	1.54	3.26	2.12
glutamine	1.49	4.18	2.81
leucine	1.45	3.13	2.17
valine	1.42	2.65	1.87
tyrosine	1.42	2.64	1.85
scyllo-inositol	1.23	12.28	9.97
homocitrulline	0.1	0.31	3.27

**Table 3:** Amino Acid Pathway.

Sub Pathway	Biochemical Name	FH/FN	NBH/NBN	AH/AN	NBN/FN	AN/FN	AN/NBN
Glycine, Serine and Threonine Metabolism	glycine	1.01	2.6	1.2	0.74	1.64	2.23
	sarcosine (N-Methylglycine)	1.23	2.76	1.13	0.54	1.06	1.96
	serine	0.95	1.66	1.36	0.86	1.42	1.65
	threonine	1.07	1.7	1.13	1.14	2.19	1.93
Alanine and Aspartate Metabolism	alanine	1.16	1.56	1.53	1.3	1.89	1.46
	N-acetylalanine	1	0.77	1.44	1.6	1	0.62
	aspartate	0.57	2.29	1.76	0.66	2.59	3.91
	asparagine	1.52	0.92	0.96	1.55	2.34	1.51
Glutamate Metabolism	glutamate	1.28	3.3	1.3	0.81	2.04	2.53
	glutamine	1.12	1.55	1.11	1.49	4.18	2.81
	gamma-aminobutyrate (GABA)	0.56	5.6	1.8	0.25	0.86	3.49
	glutamate, gamma-methyl ester	2.02	4.15	1.18	1.17	1.58	1.35
Histidine Metabolism	histidine	1.26	1.26	0.82	2.18	2.63	1.21
	1-methylimidazoleacetate	1.52	2.7	2.37	1.63	2.15	1.32
Lysine Metabolism	lysine	1.14	1.06	0.95	1.67	1.28	0.76
	2-aminoadipate	1.26	3.36	1.14	1.29	3.7	2.86
Phenylalanine and Tyrosine Metabolism	phenylalanine	1.22	1.19	0.79	1.54	3.26	2.12
	tyrosine	1.2	1.56	1.07	1.42	2.64	1.85
	p-cresol sulfate	0.65	0.85	0.7	1.09	3.23	2.97
	3,4-dihydroxyphenethyleneglycol	1.03	2.07	2.47	0.78	7.81	10.01
Tryptophan Metabolism	tryptophan	1.12	1.13	0.76	1.41	1.81	1.28
	C-glycosyltryptophan*	0.93	1.67	1.21	0.77	2.07	2.69
Leucine, Isoleucine and Valine Metabolism	leucine	1.17	1.29	0.94	1.45	3.13	2.17
	isovalerylcarnitine	1.03	7.96	1.23	0.93	2.49	2.68
	beta-hydroxyisovaleroylcarnitine	1.15	2.43	2.02	1.04	2.66	2.55
	isoleucine	1.4	1.41	1.31	1.85	2.72	1.47
	2-methylbutyrylcarnitine (C5)	0.91	2.48	0.86	0.87	1.64	1.89
	valine	1.3	1.29	1.03	1.42	2.65	1.87
	isobutyrylcarnitine	1.32	3.82	1	0.94	2.16	2.29
Methionine, Cysteine, SAM and Taurine Metabolism	methionine	1.14	1.29	1.18	1.61	3.26	2.03
	S-adenosylhomocysteine (SAH)	1.17	1.05	1.33	2.32	4.79	2.07
	2-aminobutyrate	1.56	1.54	1.72	0.23	0.86	3.71
	cysteine	1.33	0.63	0.81	1.76	3.93	2.23
	hypotaurine	0.8	7.21	2.17	1.25	4.27	3.42
	taurine	1.17	5.41	1.45	2.68	39.51	14.76
Urea cycle; Arginine and Proline Metabolism	arginine	1.09	1.38	0.95	1.23	1.7	1.38
	urea	1.37	0.73	1.64	1.13	2.87	2.55
	ornithine	1.41	0.86	1.07	1.57	1.71	1.09
	proline	0.86	1.11	1.33	1.27	0.72	0.56
	citrulline	0.71	1.86	1.16	0.96	2.2	2.29
	homocitrulline	1.56	1.8	1.82	0.1	0.31	3.27
	dimethylarginine (SDMA + ADMA)	1.14	1.21	1.52	1.02	0.5	0.49
	N-delta-acetylornithine*	0.81	1.04	1.59	0.1	0.3	3.11
	N-methyl proline	0.97	0.76	0.3	2.05	3.33	1.63
	trans-4-hydroxyproline	0.74	3	1.3	0.5	0.6	1.19
Creatine Metabolism	creatine	0.93	2.85	1.42	0.96	3.4	3.54
Polyamine Metabolism	putrescine	0.9	2.54	1.04	0.27	0.45	1.68
Glutathione Metabolism	glutathione, reduced (GSH)	1.34	32.42	1.64	0.3	58.81	196.36
	glutathione, oxidized (GSSG)	0.64	36.07	1.36	0.14	8.28	58.88
	S-lactoylglutathione	1.15	2.17	1.36	0.98	1.78	1.82
	cysteinylglycine	1.46	3.55	1.44	0.63	7.06	11.16
	5-oxoproline	3.54	1.52	1.48	1.26	1.94	1.54
	ophthalmate	1.07	1.7	0.94	0.43	5.69	13.12
	S-nitrosoglutathione (GSNO)	1	1.16	1.53	1	2.85	2.85

**Table 4:** Peptide Pathway.

Sub Pathway	Biochemical Name	FH/FN	NBH/NBN	AH/AN	NBN/FN	AN/FN	AN/NBN
Gamma-glutamyl Amino Acid	gamma-glutamylalanine	1.55	5.43	1.26	0.77	1.76	2.28
	gamma-glutamylglutamate	2.3	8.22	1.61	0.72	0.94	1.3
	gamma-glutamylmethionine	1.94	4.96	1.23	1.13	1.57	1.38
	gamma-glutamylphenylalanine	2.88	2.71	1.15	1.68	0.83	0.49
	gamma-glutamylthreonine*	1.47	4.62	1.42	0.62	1.19	1.92
Dipeptide Derivative	carnosine	0.86	3.26	1.1	0.71	0.96	1.35
Dipeptide	histidylvaline	1.02	1.27	1.11	1.44	1.89	1.31
	isoleucylglycine	0.89	0.76	0.62	4.64	10.24	2.21
	isoleucylserine	1	1.08	0.88	1.09	2.15	1.98
	leucylglutamate	1	1	0.83	1	1.2	1.2
	leucylglycine	0.85	1.21	1	1.56	3.6	2.32
	leucylserine	1	0.66	0.61	2.52	6.92	2.74
	methionylthreonine	1	1.26	1.46	1	3.23	3.23
	serylisoleucine*	1.29	1.61	1.03	1.46	1.33	0.91
	serylleucine	1.29	1.36	1.38	1.31	1.78	1.35
	serylphenyalanine	1	0.9	0.66	1.11	2.32	2.1
	serylvaline	1	0.96	1.41	1.08	1.05	0.96
	threonylglutamate	1.01	0.94	0.39	1.17	3.92	3.36
	threonylphenylalanine	1	1.04	0.89	1.04	1.55	1.49
	tyrosylglycine	1.15	0.77	1.19	2.97	1.97	0.66
	valylglycine	0.71	0.73	0.38	2.05	9.56	4.66
	valylmethionine	1	1	0.74	1	3.29	3.29
	valylserine	1	1.05	1.16	1.2	2.87	2.39
	valylthreonine	1	1	1.06	1.11	2.16	1.94

**Table 5:** Carbohydrate and Mitochondrial Metabolism.

Super Pathway	Sub Pathway	Biochemical Name	FH/FN	NBH/NBN	AH/AN	NBN/FN	AN/FN	AN/NBN
Carbohydrate	Glucose Gluconeogenesis Pyruvate Metabolism	glucose-6-phosphate (G6P)	2.35	1.59	4.66	0.91	2.14	2.36
		fructose-6- phosphate	1.59	0.88	3.96	0.95	1.94	2.04
		Isobar: fructose 1,6-diphosphate, glucose 1,6-diphosphate, myo-inositol 1,4 or 1,3-diphosphate	0.62	4.96	0.77	0.33	4.1	12.53
		3-phosphoglycerate	0.37	28.46	4.1	0.05	1.32	25.84
		phosphoenolpyruvate (PEP)	0.2	25.46	5.98	0.03	0.41	12.58
		pyruvate	1.44	6.47	3.78	0.36	4.44	12.43
		lactate	1.19	6.1	1.54	0.47	4.15	8.89
		glycerate	0.52	1.32	5.37	1.66	1.43	0.86
	Pentose Phosphate Pathway	6-phosphogluconate	1.59	31.74	3.7	0.26	4.46	17.26
		ribose 5-phosphate	1.19	2.45	2.04	0.66	6.55	9.91
		ribulose/xylulose 5-phosphate	0.54	4.35	2.2	0.35	4.18	12.09
	Pentose Metabolism	ribose	1.19	0.97	4.86	1.06	0.82	0.77
		xylitol	0.89	3.02	2.82	0.9	3.01	3.35
		arabitol	0.87	2.6	1.55	0.19	1.01	5.44
	Fructose, Mannose, and Galactose Metabolism	fructose	0.72	1.58	2.29	0.71	0.68	0.97
		sorbitol	2.25	3.19	1.59	0.98	2.15	2.2
		mannose-6-phosphate	2.41	1.94	3.85	0.82	2.34	2.87
		mannitol	0.89	2.75	2.2	0.1	0.48	4.82
		N-acetylneuraminate	1.61	1.65	1.21	1.12	1.24	1.11
		erythronate*	0.93	4.17	1.31	0.11	0.61	5.58
Energy	TCA Cycle	citrate	0.88	0.78	0.29	0.6	0.71	1.18
		fumarate	1.03	4.22	0.86	0.46	2.39	5.18
		malate	0.81	7.59	0.95	0.22	1.07	4.85
	Oxidative Phosphorylation	pyrophosphate (PPi)	0.8	6.72	2.9	0.75	5.72	7.6
		phosphate	1.02	0.85	1.22	0.86	0.87	1.01

**Table 6:** Lipid Metabolism.

Sub Pathway	Biochemical Name	FH/FN	NBH/NBN	AH/AN	NBN/FN	AN/FN	AN/NBN
Long Chain Fatty Acid	myristate (14:0)	0.9	0.46	1.2	1.91	0.56	0.29
	myristoleate (14:1n5)	0.84	0.32	0.85	3.36	0.56	0.17
	pentadecanoate (15:0)	1.4	0.6	0.82	2.28	1.1	0.48
	palmitate (16:0)	0.88	0.44	1.3	2.23	0.8	0.36
	palmitoleate (16:1n7)	0.99	0.23	1.23	2.3	0.24	0.11
	margarate (17:0)	0.87	0.3	0.95	3.58	1.46	0.41
	10-heptadecenoate (17:1n7)	0.88	0.2	0.96	2.81	0.46	0.17
	stearate (18:0)	1.03	0.46	1.16	2.18	1.18	0.54
	oleate (18:1n9)	1.14	0.2	1.23	5.96	0.47	0.08
	cis-vaccenate (18:1n7)	1.17	0.28	1.57	3.08	0.3	0.1
	nonadecanoate (19:0)	0.93	0.39	0.92	2.93	1.68	0.57
	10-nonadecenoate (19:1n9)	0.85	0.21	0.92	2.83	0.67	0.24
	eicosenoate (20:1n9 or 11)	0.99	0.27	1.27	2.52	0.46	0.18
	erucate (22:1n9)	1.02	0.33	1.24	1.82	0.29	0.16
Polyunsaturated Fatty Acid (n3 and n6)	stearidonate (18:4n3)	0.96	0.18	0.76	10.51	1.32	0.13
(n3 and n6)	eicosapentaenoate (EPA; 20:5n3)	0.42	0.12	0.78	8.14	3.02	0.37
	docosapentaenoate (n3 DPA; 22:5n3)	0.55	0.23	1.01	4.02	1.03	0.26
	docosahexaenoate (DHA; 22:6n3)	0.58	0.27	1.03	2.39	0.53	0.22
	docosatrienoate (22:3n3)	1.14	0.29	0.96	1.9	0.1	0.05
	linoleate (18:2n6)	1.18	0.41	1.97	21.83	5.58	0.26
	dihomo-linolenate (20:3n3 or n6)	1.29	0.28	1.56	11.35	2.98	0.26
	arachidonate (20:4n6)	1.43	0.39	1.21	5.02	2.02	0.4
	adrenate (22:4n6)	1.74	0.84	1.37	1.84	1.15	0.62
	docosapentaenoate (n6 DPA; 22:5n6)	1.86	0.43	1.16	3.23	0.76	0.24
	dihomo-linoleate (20:2n6)	0.93	0.32	2.26	3.07	0.52	0.17
	mead acid (20:3n9)	0.79	0.2	0.54	2.46	0.13	0.05
Fatty Acid, Branched	15-methylpalmitate (isobar with 2-methylpalmitate)	0.96	0.4	1.12	4.45	1.76	0.4
	17-methylstearate	0.74	0.31	1.33	3.37	1.07	0.32
Fatty Acid, Dicarboxylate	2-hydroxyglutarate	1	2.24	1.25	0.83	2.64	3.18
	hexadecanedioate	0.93	0.86	0.65	1.09	1.8	1.66
Fatty Acid Metabolism (also BCAA Metabolism)	butyrylcarnitine	1.06	1.93	0.77	0.98	1.03	1.05
	propionylcarnitine	0.48	3.22	1.29	0.5	1.16	2.33
Fatty Acid Metabolism	acetylcarnitine	0.81	3.8	1.48	1.6	8.66	5.4
	hexanoylcarnitine	0.98	1.44	0.87	2.01	1.16	0.58
	stearoylcarnitine	1	2.2	0.96	1.56	1.13	0.73
Carnitine Metabolism	carnitine	1.13	2.04	1.32	3.14	11.87	3.78
	3-dehydrocarnitine*	0.87	1.64	0.82	1.17	8.92	7.65
Fatty Acid, Monohydroxy	4-hydroxybutyrate (GHB)	0.28	5.58	5.74	0.14	0.6	4.26
(AcylCarnitine)	2-hydroxypalmitate	0.62	2.04	2.06	0.15	0.24	1.6
	2-hydroxystearate	0.66	2.9	2.86	0.09	0.32	3.45
Eicosanoid	5-HETE	2	0.28	1.28	6.1	0.73	0.12
Inositol Metabolism	myo-inositol	1.06	3.37	1.36	0.51	2.1	4.11
	chiro-inositol	1.12	1.44	2.14	0.57	3.29	5.77
	scyllo-inositol	1.21	3.31	0.91	1.23	12.28	9.97
	inositol 1-phosphate (I1P)	1.5	1.59	1.87	1.94	1.82	0.93
Phospholipid Metabolism	choline	1.69	0.93	1.44	2.56	1.38	0.54
	choline phosphate	0.84	1.7	1.09	0.59	1.03	1.73
	glycerophosphorylcholine (GPC)	1.76	2.19	1.2	0.74	1.27	1.71
	ethanolamine	0.71	0.67	2.04	2.41	0.89	0.37
Phospholipid Metabolism	phosphoethanolamine	0.91	8.15	1.77	0.19	1.38	7.27
Lysolipid	1-palmitoylglycerophosphocholine (16:0)	0.69	0.54	1.06	1.83	1.21	0.66
	2-palmitoylglycerophosphocholine*	0.81	0.71	1.1	1.59	1.04	0.65
	1-stearoylglycerophosphocholine (18:0)	0.86	0.62	1.07	2.04	1.99	0.98
	1-oleoylglycerophosphocholine (18:1)	1.07	0.89	0.97	1.31	1.04	0.79
	2-oleoylglycerophosphocholine*	0.92	1	0.84	0.77	0.62	0.8
	1-arachidonoylglycerophosphocholine (20:4n6)*	2.83	1.06	0.93	2.64	5.09	1.93
	2-arachidonoylglycerophosphocholine*	1.46	1.25	1.21	0.8	2.17	2.71
	1-palmitoylplasmenylethanolamine*	0.85	0.48	1.23	1.42	0.78	0.55
	1-oleoylplasmenylethanolamine*	1.14	0.41	1.27	1.71	0.47	0.27
	1-palmitoylglycerophosphoethanolamine	0.46	0.53	1.03	0.97	0.32	0.33
	2-palmitoylglycerophosphoethanolamine*	0.63	0.48	1.02	1.03	0.47	0.46
	1-stearoylglycerophosphoethanolamine	0.65	0.55	1.29	1.33	0.75	0.56
	1-oleoylglycerophosphoethanolamine	0.57	0.34	1.08	1.48	0.26	0.17
	2-oleoylglycerophosphoethanolamine*	0.74	0.59	0.93	0.95	0.32	0.34
	2-arachidonoylglycerophosphoethanolamine*	1.42	1.55	1.37	0.92	1.19	1.29
	2-docosahexaenoylglycerophosphoethanolamine*	0.83	0.71	1.17	0.73	0.5	0.68
	1-palmitoylglycerophosphoinositol*	0.59	0.57	1.03	1.41	0.97	0.69
	1-oleoylglycerophosphoinositol*	0.69	0.54	1.16	1.47	0.34	0.23
	1-stearoylglycerophosphoserine*	0.6	0.38	1.61	1.54	1.18	0.76
	1-oleoylglycerophosphoserine	0.81	0.34	1.03	2.21	0.83	0.38
	2-oleoylglycerophosphoserine*	1.04	0.62	1.28	1.81	1.01	0.56
	1-palmitoylglycerophosphoglycerol*	0.84	0.7	1.03	1.43	1.52	1.06
	1-oleoylglycerophosphoglycerol*	0.93	0.77	1.31	1.14	0.79	0.69
	2-oleoylglycerophosphoglycerol*	1.09	0.39	1.32	3.09	1	0.32
Glycerolipid	glycerol	1.17	0.87	1.51	1.35	0.88	0.66
Metabolism	glycerol 3-phosphate (G3P)	1.07	3.99	1.21	0.57	3.96	6.99
Monoacylglycerol	1-palmitoylglycerol (1-monopalmitin)	1.4	0.55	1.09	2.96	0.94	0.32
	1-stearoylglycerol (1-monostearin)	1.13	1.4	0.88	1.1	0.98	0.89
	1-oleoylglycerol (1-monoolein)	1.43	0.19	1	9.07	1.58	0.17
	1-linoleoylglycerol (1-monolinolein)	2.23	0.67	1.41	31.19	40.05	1.28
	2-linoleoylglycerol (2-monolinolein)	1.17	1.17	1.54	4.84	9.24	1.91
	1-arachidonylglycerol	1.56	0.68	0.75	4.31	7.54	1.75
	2-arachidonoyl glycerol	1.2	1.23	0.89	2.14	4.72	2.2
Sphingolipid Metabolism	stearoyl sphingomyelin	0.54	1.13	1.88	0.58	0.5	0.85
	7-alpha-hydroxycholesterol	0.68	0.23	2.9	1.7	0.24	0.14
Steroid	pregnanediol-3-glucuronide	1.53	1	1	0.16	0.16	1
Primary Bile Acid Metabolism	cholate	1	0.72	0.25	1.39	4.04	2.92

**Table 7:** Nucleotide, Cofactors, and Vitamin Metabolism.

Super Pathway	Sub Pathway	Biochemical Name	FH/FN	NBH/NBN	AH/AN	NBN/FN	AN/FN	AN/NBN
Nucleotide	Purine Metabolism Xanthine	inosine	1.27	1.97	1.48	1.33	2.78	2.09
		hypoxanthine	1.24	1.12	1.56	1.53	1.17	0.76
		xanthosine	1.06	1.24	1.51	1	2.64	2.64
	Purine Metabolism, Adenine	adenosine 5'-diphosphate (ADP)	1.43	1.39	1.03	1.14	3.18	2.78
		adenosine 5'-monophosphate (AMP)	1.23	2.35	0.69	1.06	21.37	20.12
		adenosine 3'-monophosphate (3'-AMP)	0.98	0.6	1.19	2.76	0.21	0.08
		adenosine 2'-monophosphate (2'-AMP)	1.36	0.68	0.99	2.86	1.27	0.44
		adenosine	1.17	1.86	0.78	0.78	2.22	2.84
		adenine	0.65	2.83	1.95	0.69	3.69	5.39
		cyclic adenosine diphosphate-ribose	0.77	2.78	2.34	0.53	1.2	2.29
	Purine Metabolism Guanine	guanosine 3'-monophosphate (3'-GMP)	1.1	0.7	1.11	2.45	0.26	0.11
		guanosine	1.09	1.23	1.45	1.63	1.62	0.99
		guanine	0.98	1.21	2.31	1.61	0.65	0.41
	Pyrimidine Metabolism Uracil	uridine monophosphate (5' or 3')	1	1	0.92	1	1.09	1.09
		uridine	1.3	1.35	1.36	1.52	1.42	0.93
		uracil	1.1	1.81	2.44	0.56	0.37	0.66
		pseudouridine	0.69	0.62	1.02	1.58	0.66	0.42
		3-ureidopropionate	0.34	1.96	1.52	0.28	0.28	1
		beta-alanine	0.93	3.88	1.64	0.74	2.88	3.9
	Pyrimidine Metabolism Cytidine	cytidine 5'-monophosphate (5'-CMP)	1.44	2.92	0.78	0.74	4.36	5.92
		cytidine-3'-monophosphate (3'-CMP)	1.29	1.9	1.21	1.25	0.57	0.45
		cytidine	0.99	1.28	1.86	1.46	0.83	0.57
	Purine and Pyrimidine Metabolism	methylphosphate	1.2	1.82	1.63	1.06	1.76	1.66
Cofactors and Vitamins	Nicotinate and Nicotinamide Metabolism	nicotinamide	1.49	2.78	1.96	1.02	2.28	2.23
		nicotinamide ribonucleotide (NMN)	1.19	5.33	1.92	0.27	1.07	3.97
		nicotinamide adenine dinucleotide (NAD+)	0.36	4.22	1.44	0.14	3.12	22.92
		nicotinamide adenine dinucleotide reduced (NADH)	1.39	6.72	0.44	0.52	34.33	66.3
		adenosine 5'diphosphoribose	1.09	1.53	1.47	0.84	10.73	12.7
	Riboflavin Metabolism	flavin adenine dinucleotide (FAD)	1.1	1.22	1.25	1.19	2.27	1.9
	Pantothenate and CoA Metabolism	pantothenate	1.11	2.22	2.12	0.75	0.45	0.6
	Ascorbate and Aldarate Metabolism	ascorbate (Vitamin C)	3.42	28.6	1.28	0.35	14.79	42.29
	Tocopherol Metabolism	alpha-tocopherol	1.55	1.11	1.09	2.8	8.29	2.96
	Hemoglobin and Porphyrin Metabolism	bilirubin (Z,Z)	1.92	1.26	1	1	1	1

### Changes in metabolic profile of newborn carotid arteries, as compared to fetus

As demonstrated in Table 1, a total of 86 metabolites (74 upregulated and 12 downregulated) were altered as a result of maturation from fetus to newborn. Among all the groups compared, maturation from fetus to newborn was associated with the least degree of metabolic alteration. The top metabolites (> than four-fold; P < 0.05) altered following birth in ovine carotid arteries are demonstrated in Table 8; the majority of these belong to the lipid metabolic pathway. Of note, maturation to adult (from fetus and newborn) was associated with significant downregulation in metabolites of lipid pathways (Table 6). Nonetheless, transition from fetal life to newborn had no significant effect on glutathione, tryptophan, glycine, serine, threonine (Table 3), peptides (Table 4), carbohydrate & mitochondrial (Table 5), as well as cofactors & vitamin metabolism (Table 7).

**Table 8:** Top metabolites altered in newborn carotid arteries as compared to fetus.

Biochemical Name	NBN/FN
1-linoleoylglycerol (1-monolinolein)	31.19
linoleate (18:2n6)	21.83
dihomo-linolenate (20:3n3 or n6)	11.35
stearidonate (18:4n3)	10.51
1-oleoylglycerol (1-monoolein)	9.07
eicosapentaenoate (EPA; 20:5n3)	8.14
5-HETE	6.1
oleate (18:1n9)	5.96
arachidonate (20:4n6)	5.02
2-linoleoylglycerol (2-monolinolein)	4.84
15-methylpalmitate (isobar with 2-methylpalmitate)	4.45
1-arachidonylglycerol	4.31
docosapentaenoate (n3 DPA; 22:5n3)	4.02
pregnanediol-3-glucuronide	0.16
2-hydroxypalmitate	0.15
homocitrulline	0.1
N-delta-acetylornithine*	0.1
mannitol	0.1
2-hydroxystearate	0.09

### Changes in metabolic profile of adult carotid arteries, as compared to fetus

As shown in Table 1, maturation from fetus to adult was associated with alteration of 135 metabolites (108 upregulated and 27 downregulated). The pathways most affected by this maturation were those involved in the metabolism of amino acids, peptides, and lipids. The top metabolites (> than four-fold; P < 0.05) altered in adult ovine carotid arteries, as compared to those from fetuses, are demonstrated in Table 9. The top molecules included those that play a significant role in antioxidant processes. Importantly, the long chain fatty acid pathway was significantly downregulated with maturation to adult. The pathways not affected by maturation from fetus to adult were those involved in glycine, serine, threonine, dipeptides, amino sugars, eicosanoids, phospholipids, glycerolizeds, bile acids, and panthothenate metabolism.

**Table 9:** Top metabolites altered in adult carotid arteries as compared to fetus.

Biochemical Name	AN/FN
glutathione, reduced (GSH)	58.81
1-linoleoylglycerol (1-monolinolein)	40.05
taurine	39.51
adenosine 5'-monophosphate (AMP)	21.37
ascorbate (Vitamin C)	14.79
scyllo-inositol	12.28
carnitine	11.87
isoleucylglycine	10.24
valylglycine	9.56
2-linoleoylglycerol (2-monolinolein)	9.24
3-dehydrocarnitine*	8.92
acetylcarnitine	8.66
alpha-tocopherol	8.29
glutathione, oxidized (GSSG)	8.28
3,4-dihydroxyphenethyleneglycol	7.81
1-arachidonylglycerol	7.54
cysteinylglycine	7.06
leucylserine	6.92
pyrophosphate (PPi)	5.72
ophthalmate	5.69
linoleate (18:2n6)	5.58
1-arachidonoylglycerophosphocholine (20:4n6)*	5.09
S-adenosylhomocysteine (SAH)	4.79
2-arachidonoyl glycerol	4.72
6-phosphogluconate	4.46
pyruvate	4.44
cytidine 5'-monophosphate (5'-CMP)	4.36
hypotaurine	4.27
glutamine	4.18
ribulose/xylulose 5-phosphate	4.18
lactate	4.15
palmitoleate (16:1n7)	0.24
2-hydroxypalmitate	0.24
7-alpha-hydroxycholesterol	0.24
adenosine 3'-monophosphate (3'-AMP)	0.21
pregnanediol-3-glucuronide	0.16
mead acid (20:3n9)	0.13
docosatrienoate (22:3n3)	0.1
erythritol	0.07

### Changes in metabolic profile of adult carotid arteries, as compared to newborn

This comparison demonstrated maximum change in the metabolic profile, as compared to the other experimental groups. Table 1 demonstrates that a total of 150 metabolites were altered in adult carotid arteries, as compared to those from newborn (89 upregulated and 61 downregulated). In adult, the carbohydrate and amino acid metabolic pathways were upregulated, whereas long chain, polyunsaturated, and branched fatty acid pathways were significantly downregulated, as compared to newborn. The top metabolites (> than four-fold; P < 0.05) altered in adult ovine carotid arteries as compared to those from newborn are given in Table 10. The top molecules included those, which play a significant role in antioxidant processes.

**Table 10:** Top metabolites altered in adult carotid arteries as compared to newborn.

Biochemical Name	AN/NBN	Biochemical Name	AN/NBN	Biochemical Name	AN/NBN
serine	1.65	xylitol	3.35	10-heptadecenoate	0.17
threonine	1.93	arabitol	5.44	stearate	0.54
aspartate	3.91	erythronate	5.58	oleate	0.08
glutamate	2.53	fumarate	5.18	cis-vaccenate	0.1
glutamine	2.81	malate	4.85	nonadecanoate	0.57
phenylalanine	2.12	pyrophosphate	7.6	10-nonadecenoate	0.24
tyrosine	1.85	hexadecanedioate	1.66	eicosenoate	0.18
3,4-dihydroxyphenethyleneglycol	10.01	acetylcarnitine	5.4	erucate	0.16
C-glycosyltryptophan	2.69	carnitine	3.78	stearidonate	0.13
leucine	2.17	3-dehydrocarnitine	7.65	eicosapentaenoate	0.37
beta-hydroxyisovaleroylcarnitine	2.55	4-hydroxybutyrate	4.26	docosapentaenoate	0.26
isoleucine	1.47	myo-inositol	4.11	docosahexaenoate	0.22
valine	1.87	chiro-inositol	5.77	docosatrienoate	0.05
methionine	2.03	scyllo-inositol	9.97	linoleate	0.26
S-adenosylhomocysteine	2.07	choline phosphate	1.73	dihomo-linolenate	0.26
cysteine	2.23	phosphoethanolamine	7.27	arachidonate	0.4
hypotaurine	3.42	1-arachidonoylglycerophosphocholine	1.93	docosapentaenoate	0.24
taurine	14.76	2-arachidonoylglycerophosphocholine	2.71	dihomo-linoleate	0.17
citrulline	2.29	2-linoleoylglycerol	1.91	mead acid	0.05
homocitrulline	3.27	2-arachidonoyl glycerol	2.2	15-methylpalmitate	0.4
N-delta-acetylornithine	3.11	inosine	2.09	17-methylstearate	0.32
creatine	3.54	xanthosine	2.64	hexanoylcarnitine	0.58
glutathione, reduced	196.36	adenosine 5'-diphosphate	2.78	5-HETE	0.12
glutathione, oxidized	58.88	adenosine 5'-monophosphate	20.12	choline	0.54
cysteinylglycine	11.16	adenosine	2.84	1-palmitoylglycerophosphocholine	0.66
5-oxoproline	1.54	adenine	5.39	2-palmitoylglycerophosphocholine	0.65
ophthalmate	13.12	cyclic adenosine diphosphate-ribose	2.29	1-palmitoylplasmenylethanolamine	0.55
S-nitrosoglutathione	2.85	beta-alanine	3.9	1-oleoylplasmenylethanolamine	0.27
gamma-glutamylalanine	2.28	cytidine 5'-monophosphate	5.92	1-palmitoylglycerophosphoethanolamine	0.33
gamma-glutamylthreonine	1.92	nicotinamide	2.23	2-palmitoylglycerophosphoethanolamine	0.46
isoleucylglycine	2.21	nicotinamide ribonucleotide	3.97	1-stearoylglycerophosphoethanolamine	0.56
isoleucylserine	1.98	nicotinamide adenine dinucleotide	22.92	1-oleoylglycerophosphoethanolamine	0.17
leucylglycine	2.32	nicotinamide adenine dinucleotide reduced	66.3	2-oleoylglycerophosphoethanolamine	0.34
leucylserine	2.74	flavin adenine dinucleotide	1.9	1-oleoylglycerophosphoinositol	0.23
methionylthreonine	3.23	ascorbate	42.29	1-oleoylglycerophosphoserine	0.38
serylphenyalanine	2.1	alpha-tocopherol	2.96	glycerol	0.66
threonylglutamate	3.36	hippurate	3.37	1-palmitoylglycerol	0.32
valylglycine	4.66	gluconate	1.57	7-alpha-hydroxycholesterol	0.14
valylmethionine	3.29	N-acetylalanine	0.62	hypoxanthine	0.76
valylserine	2.39	proline	0.56	adenosine 3'-monophosphate	0.08
valylthreonine	1.94	dimethylarginine	0.49	adenosine 2'-monophosphate	0.44
3-phosphoglycerate	25.84	gamma-glutamylphenylalanine	0.49	guanosine 3'-monophosphate	0.11
phosphoenolpyruvate	12.58	myristate	0.29	guanine	0.41
pyruvate	12.43	myristoleate	0.17	pseudouridine	0.42
lactate	8.89	pentadecanoate	0.48	cytidine-3'-monophosphate	0.45
6-phosphogluconate	17.26	palmitate	0.36	cytidine	0.57
ribose 5-phosphate	9.91	palmitoleate	0.11	pantothenate	0.6
ribulose/xylulose 5-phosphate	12.09	margarate	0.41	pentobarbital	0.01

Overall, as demonstrated in Tables 2, 7 metabolites showed a continuous upregulation with maturational development. Moreover, 25 metabolites were upregulated with maturation from fetus to newborn, but downregulated in the adult. Furthermore, with development from a fetus, mammals go from a relative hypoxic *in-utero* environment (arterial PaO2 ~25 Torr) to normoxic environment during newborn and adult life (arterial PaO2 ~100 Torr). Therefore, the question arises as to what extent the changes in metabolites noted above are due to development *per se* or because of the change from a relative hypoxic to normoxic environment. To answer this question, we examined metabolites in long-term hypoxic acclimatized sheep.

### Metabolic profile and long-term hypoxia exposure

Tables 3 to 7 list the biochemicals sorted by metabolic pathways in LTH animals, as compared to those that were normoxic. We conducted a Random Forest classification on the consensus of numerous decision trees. Based on the classification accuracy, it appeared that the metabolic profiles between the normoxic and LTH groups in carotid arteries isolated from newborn animals were most distinct (94% accurate). In contrast, the profiles were least distinct between the normoxic and hypoxic fetal carotid arteries (69% accurate). Markers of glucose metabolism and mitochondrial metabolism were that most significantly altered in the adult and newborn LTH groups.

### Changes in metabolic profile of fetal carotid arteries as a consequence of acclimatization to long-term hypoxia

**Fig 2:**
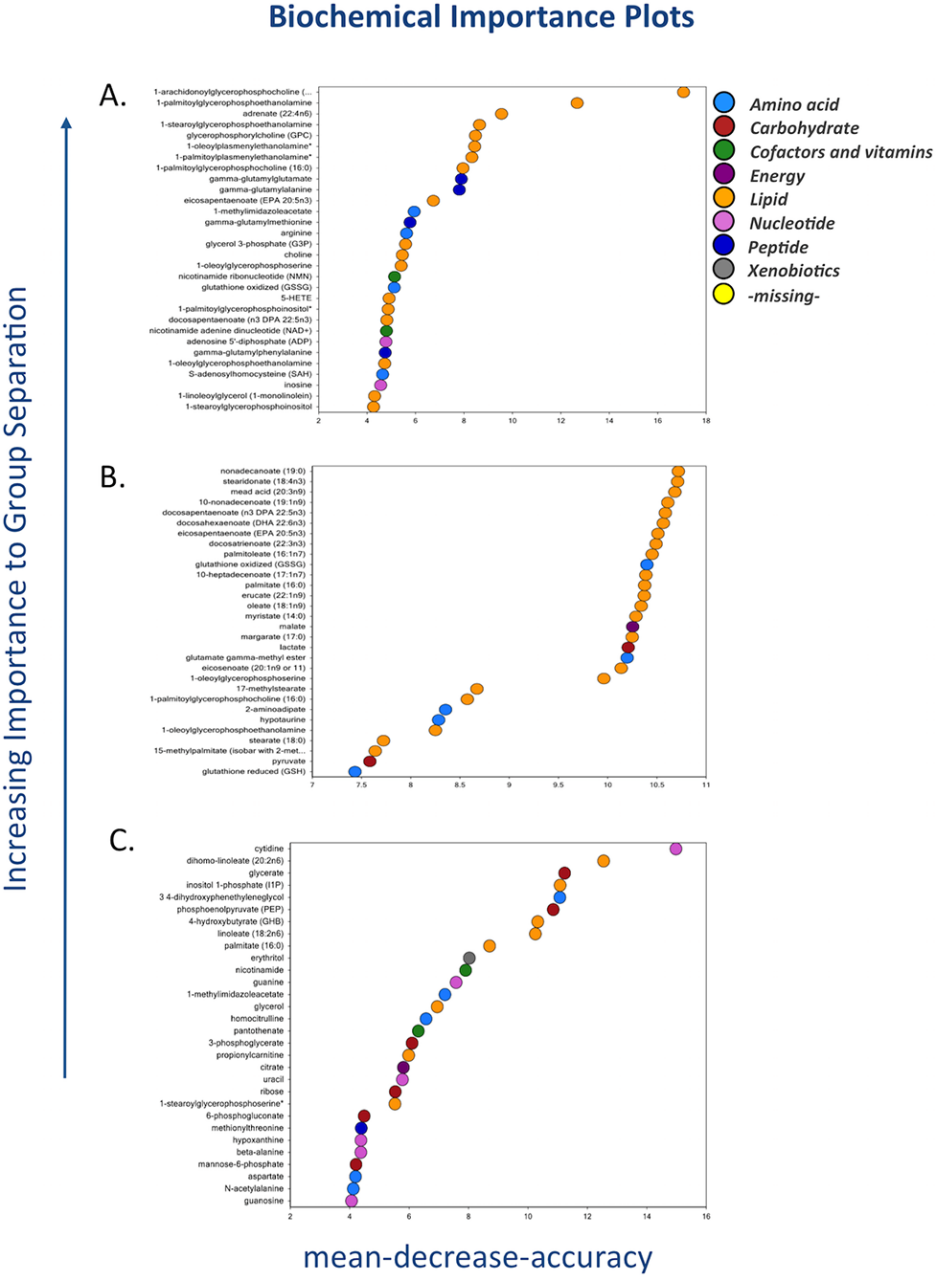
Biochemical importance plot based on Random Forest classification of metabolic profile from normoxic and hypoxic carotid arteries from (A) Fetus (B) Newborn and (C) Adults. The metabolites are plotted according to the increasing importance to group separation to elucidate the metabolic fingerprint for hypoxia in fetal carotid arteries as compared to normoxic counterpart. N = 8 in each group.

As noted above, this group showed little difference in the metabolic profile in response to LTH acclimation. Table 1 shows that out of 256 metabolites examined, only 23 were significantly altered (12 were upregulated and 11 downregulated). We observed significant changes in the biochemicals involved in membrane lipid metabolism, energy substrates & cofactors, and gamma-glutamyl amino acids. Fig 2A demonstrates the ‘Biochemical Importance Plot’ of 30 top ranking metabolites altered in response to LTH in fetal sheep carotid arteries. These molecules were predicted (Predictive Accuracy 69%) by Random Forest Classification comparing named metabolites in normoxic versus LTH near-term fetal carotid arteries. Table 11 presents the metabolites altered with long-term hypoxia. Of note, none of these metabolite met the criteria of 4-fold change.

**Table 11:** Top metabolites altered in response to LTH in fetal carotid arteries.

Biochemical Name	FH / FN	p-value
ascorbate (Vitamin C)	3.42	0.056
gamma-glutamylphenylalanine	2.88	0.002
1-arachidonoylglycerophosphocholine (20:4n6)*	2.83	0.000
mannose-6-phosphate	2.41	0.052
gamma-glutamylglutamate	2.3	0.004
1-linoleoylglycerol (1-monolinolein)	2.23	0.066
glutamate, gamma-methyl ester	2.02	0.018
gamma-glutamylmethionine	1.94	0.058
bilirubin (Z,Z)	1.92	0.001
docosapentaenoate (n6 DPA; 22:5n6)	1.86	0.033
glycerophosphorylcholine (GPC)	1.76	0.039
adrenate (22:4n6)	1.74	0.007
choline	1.69	0.035
N-acetylneuraminate	1.61	0.060
gamma-glutamylalanine	1.55	0.053
1-methylimidazoleacetate	1.52	0.099
inositol 1-phosphate (I1P)	1.5	0.067
2-arachidonoylglycerophosphocholine*	1.46	0.039
adenosine 5'-diphosphate (ADP)	1.43	0.027
isoleucine	1.4	0.032
erythritol	1.33	0.094
inosine	1.27	0.089
histidine	1.26	0.088
nicotinamide ribonucleotide (NMN)	1.19	0.069
1-palmitoylplasmenylethanolamine*	0.85	0.022
1-palmitoylglycerophosphocholine (16:0)	0.69	0.008
1-oleoylglycerophosphoinositol*	0.69	0.085
1-stearoylglycerophosphoethanolamine	0.65	0.013
2-palmitoylglycerophosphoethanolamine*	0.63	0.088
1-stearoylglycerophosphoserine*	0.6	0.035
1-palmitoylglycerophosphoinositol*	0.59	0.021
docosahexaenoate (DHA; 22:6n3)	0.58	0.034
1-oleoylglycerophosphoethanolamine	0.57	0.010
docosapentaenoate (n3 DPA; 22:5n3)	0.55	0.024
1-palmitoylglycerophosphoethanolamine	0.46	0.000
eicosapentaenoate (EPA; 20:5n3)	0.42	0.006
3-ureidopropionate	0.34	0.080

### Changes in metabolic profile of newborn carotid arteries as a consequence of LTH acclimatization

Next, we compared the metabolic profile of carotid arteries from newborn lambs from normoxic and LTH groups. This group demonstrated maximum change in the metabolic profile as a result of LTH acclimatization. As shown in Table 1, a total of 107 metabolites were altered as a result of LTH acclimatization (62 upregulated and 45 downregulated). Fig 2B illustrates significant changes in the biochemicals involved in free fatty acids, glycolysis, and glutathion/redox homeostasis. The Fig 2B demonstrates the top 30 metabolites plotted in an increasing importance to group separation as a marker of LTH newborn carotid arteries, as compared to their normoxic counterpart. The Random Forest confusion matrix predictive accuracy was 94% for this group. Table 12 lists the top metabolites altered in response to LTH exposure.

**Table 12:** Top metabolites altered in newborn carotid arteries in response to LTH.

Biochemical Name	NBH / NBN	Biochemical Name	NBH / NBN	Biochemical Name	NBH / NBN
glutathione, oxidized (GSSG)	36.07	cytidine 5'-monophosphate (5'-CMP)	2.92	2-oleoylglycerophosphoethanolamine*	0.59
glutathione, reduced (GSH)	32.42	creatine	2.85	1-palmitoylglycerophosphoinositol*	0.57
6-phosphogluconate	31.74	cyclic adenosine diphosphate-ribose	2.78	1-palmitoylglycerophosphocholine (16:0)	0.54
ascorbate (Vitamin C)	28.6	nicotinamide	2.78	1-oleoylglycerophosphoinositol*	0.54
3-phosphoglycerate	28.46	sarcosine (N-Methylglycine)	2.76	1-palmitoylglycerophosphoethanolamine	0.53
phosphoenolpyruvate (PEP)	25.46	mannitol	2.75	2-palmitoylglycerophosphoethanolamine	0.48
gamma-glutamylglutamate	8.22	gamma-glutamylphenylalanine	2.71	1-palmitoylplasmenylethanolamine*	0.48
phosphoethanolamine	8.15	1-methylimidazoleacetate	2.7	myristate (14:0)	0.46
isovalerylcarnitine	7.96	glycine	2.6	stearate (18:0)	0.46
malate	7.59	putrescine	2.54	palmitate (16:0)	0.44
hypotaurine	7.21	2-methylbutyrylcarnitine (C5)	2.48	docosapentaenoate (n6 DPA; 22:5n6)	0.43
pyrophosphate (PPi)	6.72	beta-hydroxyisovaleroylcarnitine	2.43	1-oleoylplasmenylethanolamine*	0.41
nicotinamide adenine dinucleotide reduced (NADH)	6.72	adenosine 5'-monophosphate (AMP)	2.35	linoleate (18:2n6)	0.41
pyruvate	6.47	pantothenate	2.22	15-methylpalmitate (isobar with 2-methylpalmitate)	0.4
lactate	6.1	glycerophosphorylcholine (GPC)	2.19	nonadecanoate (19:0)	0.39
gamma-aminobutyrate (GABA)	5.6	S-lactoylglutathione	2.17	arachidonate (20:4n6)	0.39
4-hydroxybutyrate (GHB)	5.58	3,4-dihydroxyphenethyleneglycol	2.07	2-oleoylglycerophosphoglycerol*	0.39
gamma-glutamylalanine	5.43	carnitine	2.04	1-stearoylglycerophosphoserine*	0.38
taurine	5.41	inosine	1.97	1-oleoylglycerophosphoserine	0.34
nicotinamide ribonucleotide (NMN)	5.33	3-ureidopropionate	1.96	1-oleoylglycerophosphoethanolamine	0.34
gamma-glutamylmethionine	4.96	butyrylcarnitine	1.93	erucate (22:1n9)	0.33
gamma-glutamylthreonine	4.62	cytidine-3'-monophosphate (3'-CMP)	1.9	dihomo-linoleate (20:2n6)	0.32
ribulose/xylulose 5-phosphate	4.35	adenosine	1.86	myristoleate (14:1n5)	0.32
fumarate	4.22	citrulline	1.86	17-methylstearate	0.31
nicotinamide adenine dinucleotide (NAD+)	4.22	methylphosphate	1.82	margarate (17:0)	0.3
erythronate	4.17	choline phosphate	1.7	docosatrienoate (22:3n3)	0.29
glutamate, gamma-methyl ester	4.15	threonine	1.7	cis-vaccenate (18:1n7)	0.28
glycerol 3-phosphate (G3P)	3.99	C-glycosyltryptophan	1.67	dihomo-linolenate (20:3n3 or n6)	0.28
beta-alanine	3.88	N-acetylneuraminate	1.65	5-HETE	0.28
isobutyrylcarnitine	3.82	3-dehydrocarnitine*	1.64	docosahexaenoate (DHA; 22:6n3)	0.27
acetylcarnitine	3.8	serylisoleucine*	1.61	eicosenoate (20:1n9 or 11)	0.27
cysteinylglycine	3.55	tyrosine	1.56	palmitoleate (16:1n7)	0.23
myo-inositol	3.37	alanine	1.56	docosapentaenoate (n3 DPA; 22:5n3)	0.23
2-aminoadipate	3.36	2-arachidonoylglycerophosphoethanolamine*	1.55	7-alpha-hydroxycholesterol	0.23
scyllo-inositol	3.31	1-stearoylglycerol (1-monostearin)	1.4	10-nonadecenoate (19:1n9)	0.21
glutamate	3.3	adenosine 5'-diphosphate (ADP)	1.39	10-heptadecenoate (17:1n7)	0.2
glycerol 2-phosphate	3.29	arginine	1.38	oleate (18:1n9)	0.2
carnosine	3.26	phosphate	0.85	mead acid (20:3n9)	0.2
propionylcarnitine	3.22	2-palmitoylglycerophosphocholine*	0.71	1-oleoylglycerol (1-monoolein)	0.19
sorbitol	3.19	1-palmitoylglycerophosphoglycerol*	0.7	stearidonate (18:4n3)	0.18
HEPES	3.1	pseudouridine	0.62	eicosapentaenoate (EPA; 20:5n3)	0.12
xylitol	3.02	pentadecanoate (15:0)	0.6	pentobarbital	0.01
trans-4-hydroxyproline	3	1-stearoylglycerophosphoethanolamine	0.55		

### Changes in metabolic profile of adult carotid arteries as a consequence of acclimatization to long-term hypoxia

Table 1 demonstrates that in adult carotid arteries as a consequence of LTH acclimatization only 39 metabolites were altered (29 upregulated and 10 downregulated). The chief biochemicals involved belong to membrane lipid metabolism, free fatty acids, glycolysis, and redox homeostasis. The top 30 metabolites suggested by the Random Forrest classification are shown in Fig 2C. The Random Forest confusion matrix predictive accuracy for the adult hypoxic carotid arteries was 81%. The top metabolites altered in adult carotid arteries in response to LTH are demonstrated in the Table 13. Similar to fetal carotid artery profile, only 7 metabolites were altered more than 4-fold (P < 0.05).

**Table 13:** Top metabolites altered in adult carotid arteries in reponse to LTH.

Biochemical Name	AH/AN	p-value	Biochemical Name	AH/AN	p-value
lauryl sulfate	8.68	0.0365	N-acetylalanine	1.44	0.0607
phosphoenolpyruvate (PEP)	5.98	0.0036	palmitate (16:0)	1.3	0.0519
4-hydroxybutyrate (GHB)	5.74	0.0009	glycerol 3-phosphate (G3P)	1.21	0.0765
glycerate	5.37	0.0008	myristate (14:0)	1.2	0.0945
ribose	4.86	0.0024	uridine monophosphate (5' or 3')	0.92	0.0162
glucose-6-phosphate (G6P)	4.66	0.0185	leucylglutamate	0.83	0.015
3-phosphoglycerate	4.1	0.0538	cysteine	0.81	0.0843
fructose-6-phosphate	3.96	0.035	serylphenyalanine	0.66	0.0255
mannose-6-phosphate	3.85	0.0215	hexadecanedioate	0.65	0.0019
pyruvate	3.78	0.0122	isoleucylglycine	0.62	0.0763
6-phosphogluconate	3.7	0.0847	leucylserine	0.61	0.0108
glycerol 2-phosphate	3.58	0.006	mead acid (20:3n9)	0.54	0.0607
pyrophosphate (PPi)	2.9	0.0839	threonylglutamate	0.39	0.0029
xylitol	2.82	0.0912	valylglycine	0.38	0.0054
3,4-dihydroxyphenethyleneglycol	2.47	0.0164	N-methyl proline	0.3	0.0082
uracil	2.44	0.0605	citrate	0.29	0.014
erythritol	2.38	0.0422	cholate	0.25	0.0428
1-methylimidazoleacetate	2.37	0.0003			
cyclic adenosine diphosphate-ribose	2.34	0.0047			
guanine	2.31	0.0939			
fructose	2.29	0.0548			
dihomo-linoleate (20:2n6)	2.26	0.0004			
mannitol	2.2	0.0335			
ribulose/xylulose 5-phosphate	2.2	0.086			
hypotaurine	2.17	0.0841			
chiro-inositol	2.14	0.0107			
pantothenate	2.12	0.0228			
ethanolamine	2.04	0.0696			
beta-hydroxyisovaleroylcarnitine	2.02	0.0328			
linoleate (18:2n6)	1.97	0.0044			
adenine	1.95	0.0149			
nicotinamide ribonucleotide (NMN)	1.92	0.0318			
stearoyl sphingomyelin	1.88	0.0515			
inositol 1-phosphate (I1P)	1.87	0.0483			
cytidine	1.86	0.0246			
homocitrulline	1.82	0.0724			
urea	1.64	0.0576			
methylphosphate	1.63	0.0174			
1-stearoylglycerophosphoserine*	1.61	0.0514			
cis-vaccenate (18:1n7)	1.57	0.0647			
dihomo-linolenate (20:3n3 or n6)	1.56	0.0537			
S-nitrosoglutathione (GSNO)	1.53	0.0156			
glycerol	1.51	0.0192			
xanthosine	1.51	0.0656			
adenosine 5'diphosphoribose	1.47	0.0924			

**Fig 3:**
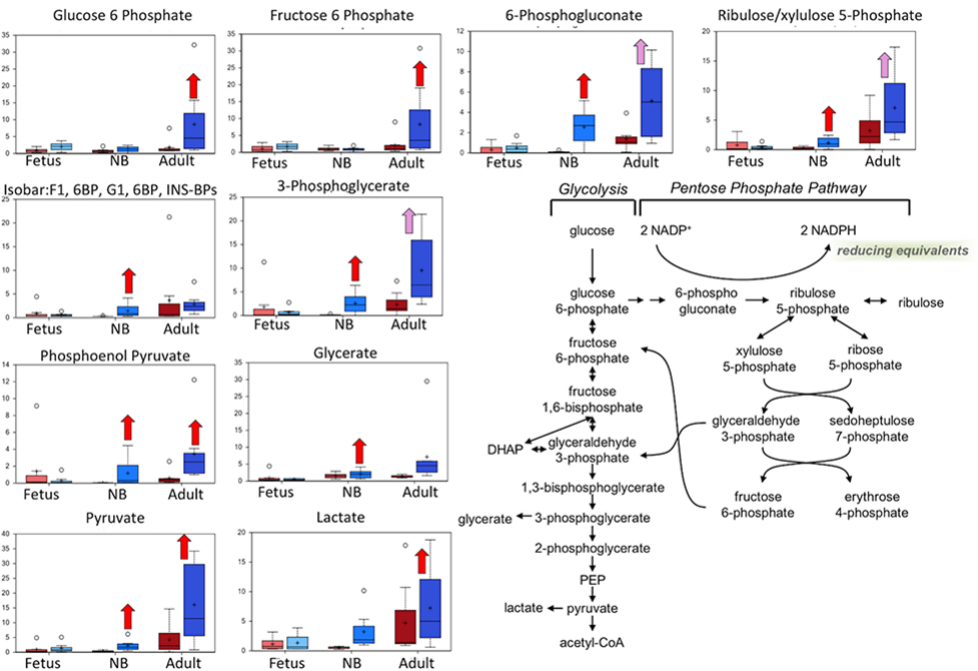
Box plots of glucose metabolism pathway comparing the six study groups In each box plots the boxes from left to right represent: normoxic and hypoxic fetus (Term); normoxic and hypoxic newborn (NB); and normoxic and hypoxic adult (Adult). The Fig also demonstrates an overview of glucose metabolism pathway. Upward red arrow means significantly (P < 0.05) higher for the noted comparison. Downward green arrow means significantly (P < 0.05) lower for the noted comparison. Upward and downward arrows together means approach significance (0.05 < P < 0.1). N = 8 in each group.

**Fig 4:**
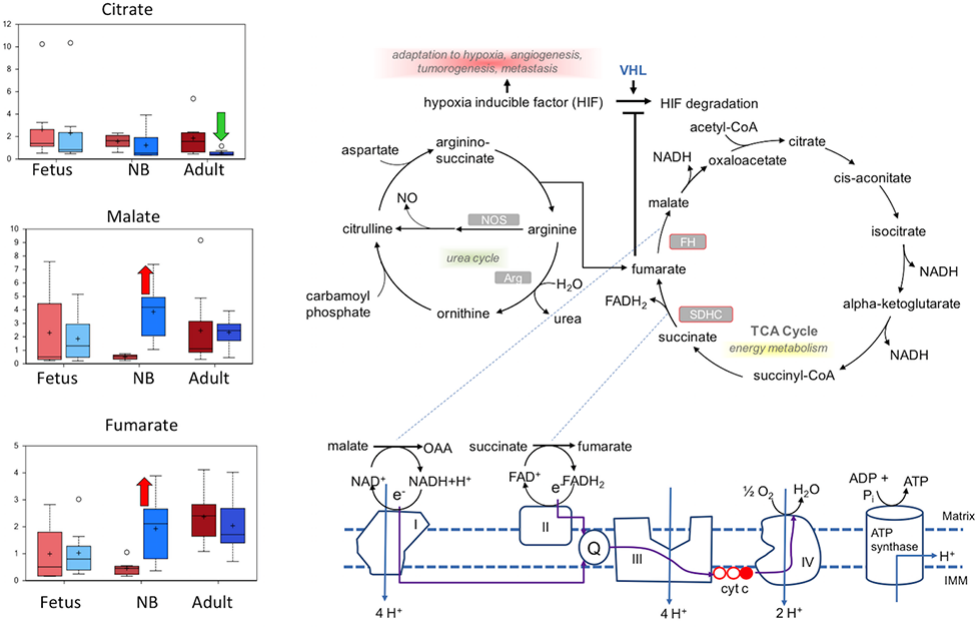
Box plots of the mitochondrial metabolism pathway comparing the six study groups. In each Fig are shown from left to right: normoxic and hypoxic fetus; normoxic and hypoxic newborn (NB); and normoxic and hypoxic adult. The Fig also demonstrates an overview of mitochondrial metabolism pathway. Upward red arrow means significantly (P < 0.05) higher for the noted comparison. Downward green arrow means significantly (P < 0.05) lower for the noted comparison. Upward and downward arrows together means approach significance (0.05 < P < 0.1). N = 8 in each group.

**Fig 5:**
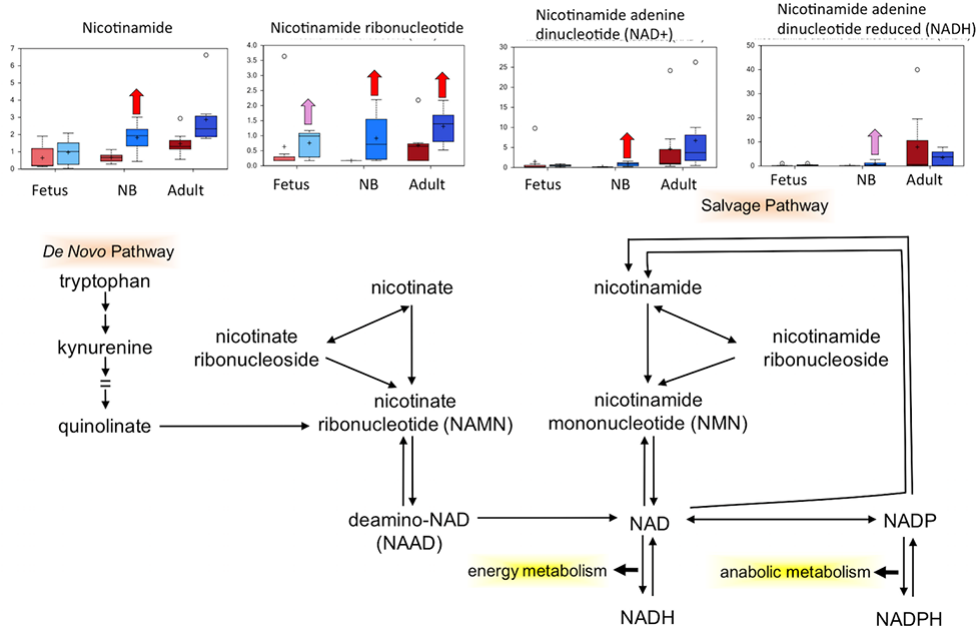
Box plots of the nicotinamide cofactor metabolism pathway comparing the six study groups. In each diagram are shown from left to right: normoxic and hypoxic fetus; normoxic and hypoxic newborn (NB); and normoxic and hypoxic adult. The Fig also demonstrates an overview of the nicotinamide cofactor metabolism pathway. Upward red arrow means significantly (P < 0.05) higher for the noted comparison. Downward green arrow means significantly (P < 0.05) lower for the noted comparison. Upward and downward arrows together means approach significance (0.05 < P < 0.1). N = 8 in each group.

**Fig 6:**
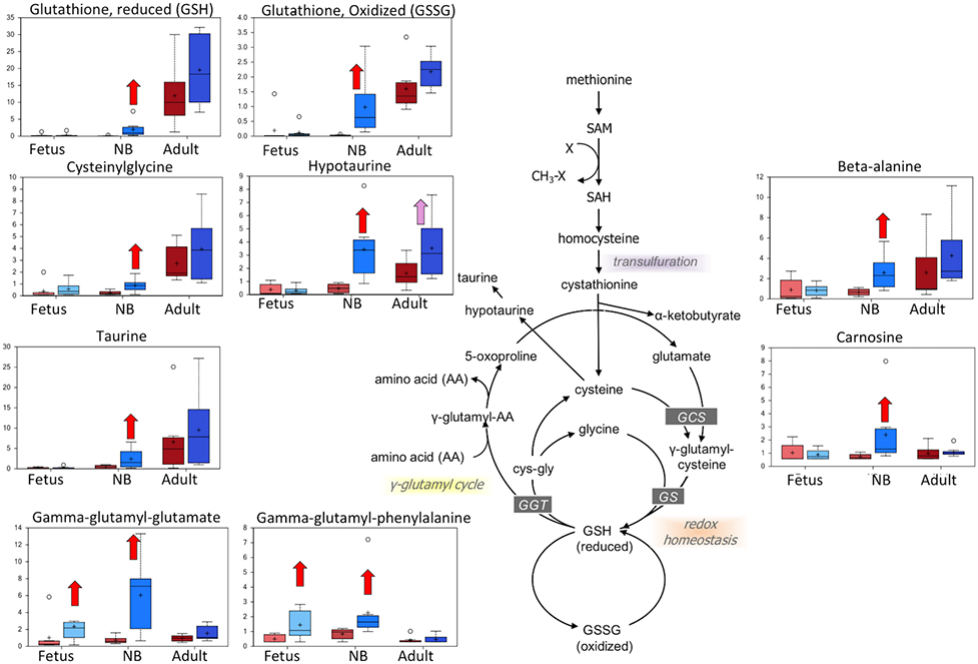
Box plots of the oxidative stress and antioxidants metabolism pathway comparing the six study groups. In each diagram are shown from left to right: normoxic and hypoxic fetus; normoxic and hypoxic newborn (NB); and normoxic and hypoxic adult. The Fig also demonstrates an overview of the oxidative stress and antioxidants metabolism pathway. Upward red arrow means significantly (P < 0.05) higher for the noted comparison. Downward green arrow means significantly (P < 0.05) lower for the noted comparison. Upward and downward arrows together means approach significance (0.05 < P < 0.1). N = 8 in each group.

**Fig 7:**
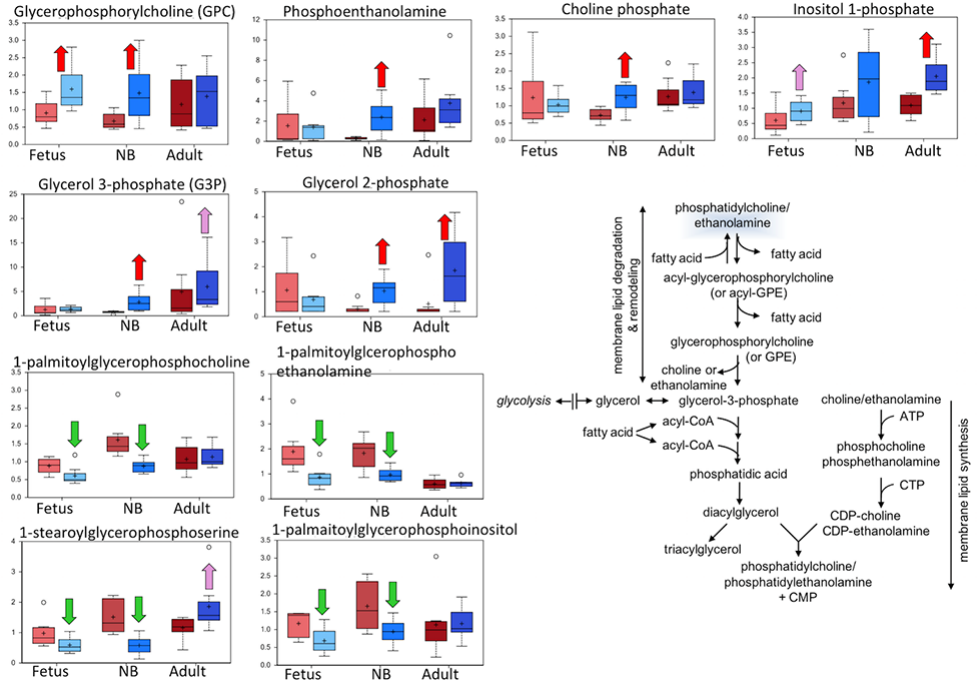
Box plots of the membrane lipid hydrolysis pathway comparing the six study groups. In each diagram are shown from left to right: normoxic and hypoxic fetus; normoxic and hypoxic newborn (NB); and normoxic and hypoxic adult. The Fig also demonstrates an overview of the membrane lipid hydrolysis pathway Upward red arrow means significantly (P < 0.05) higher for the noted comparison. Downward green arrow means significantly (P < 0.05) lower for the noted comparison. Upward and downward arrows together means approach significance (0.05 < P < 0.1). N = 8 in each group.

**Fig 8:**
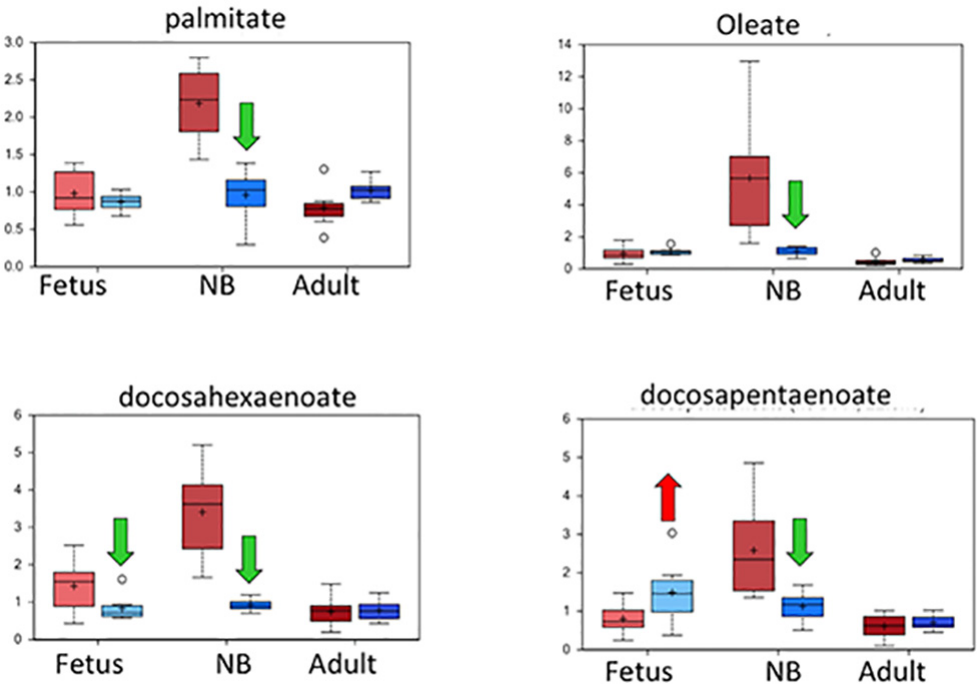
Box plots of the free fatty acid metabolism pathway comparing the six study groups. In each diagram are shown from left to right: normoxic and hypoxic fetus; normoxic and hypoxic newborn (NB); and normoxic and hypoxic adult. The Fig also demonstrates an overview of the free fatty acid metabolism pathway. Upward red arrow means significantly (P < 0.05) higher for the noted comparison. Downward green arrow means significantly (P < 0.05) lower for the noted comparison. Upward and downward arrows together means approach significance (0.05 < P < 0.1). N = 8 in each group.

Overall, Table 14 shows that only 3 molecules were altered with significance value of P < 0.05 in response to LTH in all the three groups examined. Figs 3–8 demonstrate that the major pathways regulated by LTH were glucose metabolism, mitochondrial metabolism, nicotinamide cofactor metabolism, oxidative stress and antioxidants, membrane lipid hydrolysis, and free fatty acid metabolism.

**Table 14:** Metabolites altered in all three development groups as a consequence of hypoxia.

Biochemical Name	FH/FN	NBH/NBN	AH/AN
1-methylimidazoleacetate	1.52	2.7	2.37
nicotinamide ribonucleotide (NMN)	1.19	5.33	1.92
homocitrulline	1.56	1.8	1.82
glucose-6-phosphate (G6P)	2.35	1.59	4.66
sorbitol	2.25	3.19	1.59
6-phosphogluconate	1.59	31.74	3.7
mannose-6-phosphate	2.41	1.94	3.85
gamma-glutamylglutamate	2.3	8.22	1.61
1-stearoylglycerophosphoserine	0.6	0.38	1.61
adenine	0.65	2.83	1.95
lauryl sulfate	0.62	0.63	8.68
gamma-aminobutyrate (GABA)	0.56	5.6	1.8
3-phosphoglycerate	0.37	28.46	4.1
phosphoenolpyruvate (PEP)	0.2	25.46	5.98
ribulose/xylulose 5-phosphate	0.54	4.35	2.2
4-hydroxybutyrate (GHB)	0.28	5.58	5.74
3-ureidopropionate	0.34	1.96	1.52

## Discussion

Among the “-omics” fields of contemporary research, that of metabolomics is helping to define the cellular phenotype of metabolites that specify function. The concept that a metabolic profile or fingerprint can define specific cellular functions, while relatively new, is helping to advance our understanding of many aspects of biology including health and disease [1]. In the present report using the cranial carotid artery as a case study for major determinant of blood flow to the brain, we observed significant differences between the near-term fetus and the newborn lambs, and between these and the adult. In contrast, values for those animals acclimatized to high altitude, long-term hypoxia showed greater variability, but only relatively minor differences as compared to the normoxic controls for a given age group.

The exposure of LTH to fetus resulted in a minor change in metabolic profile indicating the ability of sheep to undergo successful acclimatization. Of importance, successful acclimatization to LTH involves subtle changes in the vasculature to meet differential oxygen needs of the various organs. Accumulating evidence suggests that prolonged hypoxia leads to epigenetic changes, which in turn lead to changes in gene expression [41]. These change in gene expression are the major factor in LTH-induced phenotypic and physiological changes for successful hypoxic acclimatization. However, the mechanistic link between LTH and epigenetic changes which leads to successful acclimatization remain incompletely understood.

During the past decade, evidence has demonstrated that the changes in cellular metabolism may be a major factor in the epigenetic regulation of gene expression to adapt to the stress of LTH [34,41]. Moreover, LTH-mediated changes in phenotype and transcriptome are differentially regulated in the developing fetus, as compared to the adult [24,31,38]. For example, LTH promotes age-dependent smooth muscle cell remodeling that leads to larger arteries in the fetus but smaller arteries in the adult [42]. Thus, the question arises, to what extent does hypoxia differentially alter the metabolism in fetus, newborn, and adult vasculature? The present study confirms that acclimatization to LTH leads to differential regulation of metabolism with developmental age.

### Markers of glucose metabolism and mitochondrial metabolism were altered in the adult and newborn LTH groups

In the present study, we observed that the markers of glucose metabolism and mitochondrial metabolism were altered significantly in the adult and newborn LTH groups (Fig 3). Intermediates and derivatives of glycolysis, including glucose-6-phosphate, 3-phosphoglycerate, pyruvate, glycerate, and lactate, were elevated in newborn and/or adult carotid samples isolated from animals subjected to LTH. As shown in Table 1 and Fig 2B, there was a concurrent increase in fumarate and malate associated with the mitochondrial tricarboxylic acid (TCA) cycle in newborn carotid artery samples, whereas citrate was significantly decreased in the adult LTH group relative to the adult normoxic group (Fig 4). Interestingly, the build-up of fumarate due to restricted activity of the mitochondrial respiration chain under oxygen-deficient conditions has been linked to increased stabilization of hypoxia inducible factor via the fumarate inhibition of prolyl-4-hydroxylases [43]. These changes indicate that changes in the metabolic profile of carotid arteries from newborn animals were significantly different, as compared to fetus or adult. Although the adult hypoxic carotid arteries did not display a build-up of fumarate or malate, it is possible that the accumulation of glycolytic intermediates and pentose phosphate pathway intermediates, such as 6-phosphogluconate and ribulose-5-phosphate/xylulose-5-phosphate, represented a subtle redirection of glucose carbons away from mitochondrial consumption via the TCA cycle. In the last two decades, changes in glucose metabolism under aerobic to anaerobic (hypoxic) condition has been implicated in a number of disorders including cancer, obesity, cardiovascular, cerebral, and renal disorders [44–48]. Moreover, hypoxic adaptation has been implicated to play a key role in neurodegenerative disorders [49,50]. The findings of the present study suggest differential capacity of fetus, newborn, and adult to metabolize glucose and adapt under hypoxic conditions. Further *in-vitro* studies on cells obtained from these three different age group with hypoxia exposure are needed to investigate the mechanisms of these developmentally regulated metabolic changes.

### Nicotinamide metabolism may reflect mitochondrial stress

As illustrated in Fig 2A, nicotinamide, and its derivatives nicotinamide mononucleotide (NMN), nicotinamide adenine dinucleotide (NAD+), and nicotinamide adenine dinucleotide hydrate (NADH), were elevated in carotid arteries isolated from newborn animals subjected to LTH, whereas only NMN was elevated in the other LTH age groups (Fig 5). Nicotinamide and NMN are constituents of the salvage pathway of NAD+ synthesis that recycles nicotinamide contained in NADP+ and NADPH back to NAD+ (Fig 5). Of importance, nicotinamide has been reported to confer protection from mitochondrial stress following hypoxia [51, 52]. Thus, it is possible that the increased levels of nicotinamide and its derivatives was a further indication of LTH having a greater impact on newborn carotid arteries, in comparison to those isolated from fetuses or adults. Importantly, nicotinamide has recently been shown to improve neuronal function following sever hypoxia [53,54] and anti-nicotinamide treatment has been linked with severe neurotoxicity [55]. Thus, increased nicotinamide in the present study may represent an important adaptive mechanism.

### Hypoxia increased cysteine-derived compounds in carotid arteries isolated from newborns

Metabolites derived from the sulfur-containing amino acid cysteine, such as taurine and glutathione, were elevated in newborns born to high altitude-acclimated ewes. Gamma-glutamyl amino acids, which are synthesized by gamma-glutamyl transpeptidase (GGT), were elevated in hypoxic fetuses and newborns with respect to their normoxic reference controls. Elevated circulating levels of GGT activity can be a sign of liver inflammation, but in addition to a detoxification role played in the liver, these enzymes facilitate the energy-driven import of amino acids into tissues. Finally, endogenous antioxidants—taurine and carnosine—as well as respective precursors, hypotaurine and beta-alanine, were elevated in the hypoxic newborn samples, but not in other groups with respect to their normoxic controls. The higher relative levels of glutathione and antioxidants in hypoxic newborn carotid samples could reflect higher levels of oxidative stress in this group, but the other age groups under LTH did not share this response (Fig 6).

### Markers of complex lipid metabolism distinguished between normoxic and hypoxic groups

Metabolites indicating the hydrolysis of phosphatidylcholines, phosphatidylglycerol [56], and phosphatidylinositol-1-phosphate—namely, glycerophosphocholine (GPC), glycerol-2-phosphate (G2P), and inositol-1-phosphate (I1P)—were elevated in at least one of the age groups acclimated to high altitude relative to their normoxic controls (Fig 7). Glycerol-3-phosphate (G3P), choline phosphate, and phosphoethanolamine, which are potential markers of phospholipid degradation but also can serve as substrates for phospholipid synthesis, also were elevated in the hypoxic newborn carotid arteries. A range of lysophospholipids containing an acyl group at the sn-1 position on the glycerol backbone—for example 1-palmitoylglycerophosphocholine and 1-stearolyglycerophosphoserine—were decreased in the LTH acclimatized newborns, and, to a lesser extent in the fetuses. Lysolipids with the sn-1 configuration are the products of phospholipase A2 (PLA2) enzymes whereas phosphoethanolamine, choline phosphate, and I1P are potential products of phospholipase C-class enzymes. These results suggest that LTH altered complex lipid metabolism; however, the balance among membrane phospholipid degradation, remodeling, and synthesis could not be judged from the metabolic profile alone. Of importance, hypoxia can lead to imbalance of liver lipid homeostasis and is a risk factor for non-alcoholic fatty liver diseases and steatohepatitis [57]. Thus, hypoxia induced changes in lipid metabolism in carotid arteries may further lead to atherosclerosis and stiffening.

### Free fatty acids were strong differentiators of the newborn group

Saturated, monounsaturated, and polyunsaturated long-chain free fatty acids (FFAs) differed significantly between the normoxic and LTH newborn carotid samples (Fig 8). The pattern of changes appeared as an increase of free fatty acids in the normoxic newborns compared to normoxic fetal and adult samples, whereas the levels of FFAs among the different hypoxic age groups were similar. Fatty acids become the primary fuel for the heart following birth [58,59] and hypoxia has been reported to decrease the activity of lipases that release free fatty acids from circulating triglycerides [60,61]. Thus it is possible that hypoxia similarly limits the supply of free fatty acids to the carotid arteries.

### Physiological Relevance and Clinical Perspective

Of all the stresses to which the fetus and newborn infant are subjected, perhaps the most important and clinically relevant is that of hypoxia. Experimentally produced hypoxia has also proved a useful tool with which to explore fundamental aspects of systems and cellular regulatory mechanisms. In general, the basic mechanisms whereby an organism adapts to high-altitude, long-term hypoxemia are unknown. In a manner similar to stresses such as hypovolemia and hyperthermia, hypoxia stimulates a variety of systemic and cell/tissue specific hormonal and molecular homeostatic responses in the organism. Additionally, LTH is of fundamental physiologic importance in its own right, and has great clinical relevance. For instance, in pregnant women at high altitude, the incidence of toxemia of pregnancy (preeclampsia) is elevated, as compared with normoxic controls. In addition to intrauterine growth restriction (which occurs in 7 to 10% of all pregnancies and is particularly common at high altitude), LTH is associated with serious problems such as dysregulation of cerebral blood flow with attendant intraventricular and germinal matrix hemorrhage, making them subject to neurological and other developmental handicaps [62–65], as well as persistent fetal circulation, persistent pulmonary hypertension of the newborn, necrotizing enterocolitis, and other conditions associated with increased perinatal morbidity. The present study was an attempt to identify metabolic changes accompanying LTH induced acclimatization. This study, thus lays the foundation of in depth mechanistic analysis of the identified metabolites in hypoxia-induced physiological and pathological changes.

## Conclusions

This global metabolic profiling study on carotid arteries was conducted to understand in more detail the adaptive responses to LTH in full term fetuses, newborns, and adult sheep. Random Forest classification was able to classify between normoxic and LTH within an age group with high accuracy. The metabolic profile between the normoxic and LTH groups in arteries isolated from newborn animals was most distinct (94% accurate), while the least distinction was between the normoxic and hypoxic fetal carotid arteries (69% accurate). Perhaps this is because the fetus develops in an already hypoxemic state, as compared to the newborn or adult [66,67]. Moreover, free fatty acids, glutathione & redox homeostasis markers, glycolysis metabolites, membrane lipid metabolism indicators, and gamma-glutamyl amino acids were highly represented among the Random Forest compounds key to group separation. Based on the build-up of compounds associated with glycolysis, the pentose phosphate pathway, and the mitochondrial tricarboxylic acid cycle in the carotid arteries collected from newborn animals subjected to LTH, we suggest that this age group, relative to the fetal and adult, experienced greater mitochondrial stress in response to LTH. The proposed greater hypoxic stress in newborns was bolstered by observations of increased nicotinamide-containing compounds. These may be an adaptation to maintain NAD+ and NADH homeostasis, as well as greater levels of glutathione metabolism and endogenous antioxidants, in the face of a relative oxygen deficiency. LTH appeared to alter complex lipid metabolism in all groups whereas free fatty acids, which were elevated in newborn carotids under normoxic conditions, tended to be similar across all LTH age groups. Overall, the present study identified a significant change in metabolism as a consequence of both maturational age and an acclimatization response to prolonged hypoxia.

## Supporting Information

S1 File Description of Quality Control of the Samples (Table A). Standards used for Quality Control (Table B). Heat map of statistically significant biochemicals profiled in this study (Table C).
(XLSX)
